# A Treatise on Diseases of the Nervous System

**Published:** 1905-08

**Authors:** 


**A Treatise on Diseases of the Nervous System.** By **L. Harrison Mettler,** M.D., Associate Professor of Neurology, College of Medicine of the University of Illinois. Octavo, 1,000 pages. Illustrated, including two color plates. Chicago: Cleveland Press. 1904. (Cloth, $5.00; half morocco, $6.00.)

The appearance of an exhaustive treatise on nervous diseases, such as this author has produced, is evidence of the great interest taken and still greater energy displayed in this branch of medical science. It may be due in some measure to the newer pathology based on the neuron doctrine and an effort to acquire a better conception of modern neurology through a better understanding of the neuronic structure of the nervous system. Although much is known and comprehended as regards the neuron, still much more remains to be learned and when all is said, it must be admitted that this doctrine and its teachings has done more to illuminate the dark places of neurology than any other single scientific generalisation heretofore promulgated.

This volume is written with the great object constantly in view of educating the theories and laws of nerve action, through the medium of the neuron. Sections A and B are devoted to a general consideration of neurology, the peculiarities of the nervous system, the etiology, pathology, the symptomatology, and treatment of nervous diseases. Under treatment the author takes up certain phases not usually met with in textbooks, such as (1) the prevention of the disease itself, its return, or its exacerbation should always be attempted by the aid of proper hygienic and prophylactic measures. Under this head the author advises about the marriage and propagation of individuals of neuropathic tendencies, proper exercises, diet, baths, and environment; (2) every endeavor should be made to remove or render inactive the cause; (3) the symptoms should be controlled in the hope of thereby removing their cause, or at least of mitigating the distress produced by them.

Massage, Swedish movements, electrotherapy, and climatotherapy are all briefly discussed and practical points given as to their administration. The reader at once gains one point in the author's handling of this chapter, and that is his practicability and good judgment in the general consideration of treatment of these diseases.

Section B is devoted to the consideration of the neuronic diseases, and he offers the following classification: the functional neuronic diseases, (1) those of the cerebrospinal system; (2) those of the sympathetic system. The organic neuronic diseases: (1) those of the afferent system; (2) those of the efferent system; (3) those of the efferent and afferent systems.

In describing the functional neuronic diseases the author well says, "All disease must be functional in its incipiency. Some diseases remain functional; others go on to cause slight or minute changes undiscoverable by our present means of investigation; while still others advance to the production of gross microscopic and macroscopic alterations, much destruction, and even annihilation of tissue."

The consideration of hysteria is exhaustive and most interesting. The labors of Charcot and the school of the Salpetriere are given full credit. In the treatment the author says, "the guiding thought should always be its psychic nature," and mental discipline accordingly invoked.

The chapters on epilepsy, chorea, neurasthenia and the tics are interesting reading, coupled with good sound reasoning and judgment. Of the neuronic organic diseases, the author deals quite fully with the muscular atrophies, locomotor ataxia and the paraplegias. Section C is devoted to the non-neuronic diseases, such as the tumors and other destructive processes of the spinal cord and brain. Section D treats of general maladies with leading neurological symptoms, such as alcoholism, the opium and kindred habits, tetanus, hydrophobia, arthritis deformans, and other toxic affections.

Taken as a whole, Mettler's treatise is a very creditable exposition of American neurology and will certainly find favor in the profession. It is exhaustive and still interesting, bringing down neurological facts and theories to the present moment in a manner deserving the greatest credit. W. C. K.

